# Microwave Ablation Versus Radiofrequency Ablation for Hepatocellular Carcinoma: A Systematic Review and Meta-Analysis

**DOI:** 10.7759/cureus.117078

**Published:** 2026-09-28

**Authors:** Lucas Ortiz, Kevin Vasquez, Juan M Lozano, Andres Gonzalez

**Affiliations:** 1 Interventional Radiology, Fundación Universitaria Sanitas, Bogotá, D.C., COL

**Keywords:** hepatocellular carcinoma, interventional radiology, local tumor progression, meta-analysis, microwave ablation, radiofrequency ablation, systematic review, thermal ablation

## Abstract

Microwave ablation (MWA) and radiofrequency ablation (RFA) are established curative-intent treatments for early-stage hepatocellular carcinoma (HCC), but their relative benefits remain uncertain. We systematically synthesized comparative evidence on the effectiveness and safety of MWA versus RFA in adults with HCC. PubMed/Medical Literature Analysis and Retrieval System Online (MEDLINE), Web of Science, Scopus, SciELO, and Latin American and Caribbean Health Sciences Literature (LILACS) were searched through March 2026. Study eligibility, extraction of treatment-specific data, and methodological quality assessment were independently evaluated by two reviewers. Quantitative synthesis used random-effects models, with risk ratios (RRs) for dichotomous endpoints and hazard ratios (HRs) for time-to-event endpoints. Confidence in the evidence for each outcome was judged according to the Grading of Recommendations Assessment, Development and Evaluation (GRADE) framework. Thirty-eight studies, including seven randomized controlled trials, contributed 8,347 participants. Initial complete ablation was similar with MWA and RFA (28 studies; 6,036 participants; RR 1.01, 95% confidence interval (CI) 1.00-1.02; I²=0%). MWA was associated with less local tumor progression (27 studies; 5,166 participants; RR 0.67, 95% CI 0.54-0.83; I²=54%), with a consistent association in randomized trials (RR 0.55, 95% CI 0.40-0.76; I²=0%). No conclusive between-technique differences were found for intrahepatic distant recurrence, overall survival, recurrence-free survival, major or minor complications, or periprocedural mortality. No statistically conclusive difference in initial complete ablation was demonstrated between MWA and RFA. MWA may provide better local control, although current evidence does not show a corresponding survival benefit. Treatment choice should incorporate tumor location, the expected ablation margin, procedural safety, operator experience, technology availability, and cost.

## Introduction and background

Primary liver cancer remains a major cause of cancer mortality, and hepatocellular carcinoma (HCC) represents its predominant histologic type [1,2]. Within the Barcelona Clinic Liver Cancer (BCLC) 2026 framework, ablative therapy is among the potentially curative treatments considered for very early- and early-stage HCC (BCLC 0/A) patients, together with resection and transplantation, selected according to tumor burden, liver function, transplant eligibility, technical feasibility, and patient-specific factors [3,4]. Its role becomes particularly relevant when surgery is unsuitable because of limited hepatic reserve or when transplantation is not feasible, allowing curative-intent treatment of selected small tumors [5,6].

Radiofrequency ablation (RFA) has long served as the reference thermal technique. Alternating electrical current produces ionic agitation and tissue heating around the electrode, but the resulting ablation zone depends partly on conductive heat transfer. Blood flow in nearby vessels can remove heat from the target, creating the heat-sink effect and reducing treatment efficacy in perivascular tumors [7,8].

Microwave ablation (MWA) creates an electromagnetic field that produces rapid volumetric heating through molecular rotation and ionic polarization. In theory, this mechanism permits higher intratumoral temperatures, larger treatment zones, shorter application times, and less susceptibility to vascular heat loss than RFA [9,10]. Contemporary consensus recommendations nevertheless regard both modalities as acceptable first-line ablative treatments for early-stage HCC [11].

Earlier meta-analyses of randomized evidence generally reported comparable complete response and survival between the modalities, although some suggested improved disease control with MWA [12,13]. The overall evidence remains difficult to interpret because studies differ substantially in technology, procedural technique, tumor characteristics, follow-up, and endpoint definitions, with observational designs accounting for a large proportion of the available comparisons.

We therefore performed an updated systematic review and meta-analysis of direct MWA-RFA comparisons in adults with HCC. The review evaluated initial complete ablation, local and distant recurrence, survival, complications, and periprocedural mortality; applied design-specific risk-of-bias tools; and rated outcome-level certainty using the Grading of Recommendations Assessment, Development and Evaluation (GRADE) framework.

## Review

Methods

Protocol and Registration

Reporting was guided by the Preferred Reporting Items for Systematic Reviews and Meta-Analyses (PRISMA) 2020 recommendations [14]. Before completion of the review, the protocol was registered with the International Prospective Register of Systematic Reviews (PROSPERO; CRD420261295141), where the research question, eligibility criteria, outcomes, and planned evidence-synthesis methods were prespecified. Analyses involving perivascular or subcapsular location, previous treatment status, baseline alpha-fetoprotein concentration, and liver disease etiology were subsequently added as exploratory post hoc analyses.

Eligibility Criteria

Study eligibility was structured around prespecified population, intervention, comparator, outcome, and study-design domains. We included adults aged 18 years or older with HCC diagnosed by clinical, imaging, or histopathologic criteria. Imaging-based diagnosis could follow the American Association for the Study of Liver Diseases, European Association for the Study of the Liver, or Liver Imaging Reporting and Data System criteria [3,15,16]. Most participants had BCLC stage 0 or A disease, although selected stage B populations suitable for ablation were eligible.

Inclusion required a head-to-head comparison of MWA and RFA within a randomized study or comparative observational cohort. Ablation performed percutaneously, laparoscopically, or through an open approach was eligible. Eligible designs comprised randomized controlled trials and nonrandomized comparative investigations, including prospective and retrospective cohorts, case-control designs, propensity score-matched cohorts, and analyses applying inverse probability of treatment weighting.

Outcomes of interest were initial complete ablation, local tumor progression, intrahepatic distant recurrence, extrahepatic recurrence, overall survival, recurrence-free survival, major complications, minor complications, and periprocedural mortality. Noncomparative studies, preclinical or simulation studies, reports without separately extractable HCC results, duplicate or overlapping reports, systematic reviews, and previous meta-analyses were excluded. Publications had to be in English, Spanish, or Portuguese and dated 2005 or later. For recurrence and survival outcomes, at least 12 months of follow-up and 20 participants per treatment arm were required. The complete criteria are presented in Table 1.

**Table 1:** Eligibility criteria based on the Population, Intervention, Comparator, Outcomes, and Study Design Framework

Component	Eligibility criterion
Population	Adults aged 18 years or older with hepatocellular carcinoma (HCC) diagnosed by clinical, imaging, or histopathologic criteria. Imaging-based diagnosis could follow the American Association for the Study of Liver Diseases (AASLD), European Association for the Study of the Liver (EASL), or Liver Imaging Reporting and Data System (LI-RADS) criteria. Most participants had Barcelona Clinic Liver Cancer (BCLC) stage 0 or A disease; selected stage B populations suitable for ablation were eligible.
Intervention	Microwave ablation (MWA) performed through a percutaneous, laparoscopic, or open approach.
Comparator	Radiofrequency ablation (RFA) directly compared with MWA within the same randomized trial or comparative cohort.
Outcomes	Initial complete ablation; local tumor progression; intrahepatic distant recurrence; extrahepatic recurrence; overall survival; recurrence-free survival; major complications; minor complications; and periprocedural mortality.
Study designs	Randomized controlled trials and comparative nonrandomized studies, including prospective or retrospective cohorts, case-control studies, propensity score-matched analyses, and inverse probability of treatment-weighted analyses.
Exclusion criteria	Noncomparative studies; preclinical or simulation studies; reports without separately extractable HCC results; duplicate or overlapping reports; systematic reviews; and previous meta-analyses.
Restrictions	Publications in English, Spanish, or Portuguese; publication year 2005 or later; and, for recurrence and survival outcomes, at least 12 months of follow-up and at least 20 participants per treatment arm.

Information Sources and Search Strategy

Searches were performed in PubMed/Medical Literature Analysis and Retrieval System Online (MEDLINE), Web of Science, Scopus, SciELO, and Latin American and Caribbean Health Sciences Literature (LILACS), covering each database from its inception to March 2026. Database-specific queries combined indexed terminology with free-text expressions describing HCC, MWA, and RFA. The electronic search was supplemented by citation searching of eligible reports and relevant reviews. Complete database-specific strategies are supplied in the supplementary material.

Study Selection

Once duplicate records had been removed, two investigators evaluated titles and abstracts independently. Publications considered potentially eligible were subsequently examined in full text by both reviewers using the predefined inclusion and exclusion criteria. Differences in eligibility judgments were first discussed between the reviewers and were referred to a third investigator when consensus could not be reached. The specific basis for exclusion was documented for each report removed at the full-text stage. The excluded full-text reports and their reasons are listed in Table 2 [17-35].

**Table 2:** Reports excluded after full-text assessment and reasons for exclusion

Excluded report	Reason for exclusion
Chinnaratha et al., 2016 [17]	Systematic review and meta-analysis (secondary research).
Dou et al., 2022 [18]	Systematic review and meta-analysis (secondary research).
Facciorusso et al., 2016 [19]	Systematic review and meta-analysis (secondary research).
Facciorusso et al., 2020 [12]	Systematic review and meta-analysis (secondary research).
Francica et al., 2016 [20]	Did not provide the required direct microwave ablation (MWA)-radiofrequency ablation (RFA) comparison within the eligible analytic cohort.
Han et al., 2020 [21]	Systematic review and meta-analysis (secondary research).
Huo and Eslick, 2015 [22]	Systematic review and meta-analysis (secondary research).
Shi et al., 2014 [23]	Compared microwave ablation (MWA) with surgical resection rather than radiofrequency ablation (RFA).
Khan et al., 2021 [24]	Systematic review and meta-analysis (secondary research).
Miyamoto et al., 2021 [25]	Both treatment groups received previous transarterial therapy; the report did not provide the prespecified direct ablation-only comparison.
Rey and Darmadi, 2024 [26]	Systematic review and meta-analysis (secondary research).
Ricci et al., 2020 [27]	Review article (secondary research).
Shaqran et al., 2024 [28]	Systematic review and meta-analysis (secondary research).
Spiliotis et al., 2021 [29]	Systematic review and meta-analysis (secondary research).
Tan et al., 2019 [30]	Systematic review and meta-analysis (secondary research).
Huang et al., 2016 [31]	Letter/commentary rather than a primary comparative study.
Yu et al., 2021 [13]	Systematic review and meta-analysis (secondary research).
Zhang et al., 2023 [32]	Systematic review and meta-analysis (secondary research).
Zhang et al., 2014 [33]	Preclinical experimental study rather than a comparative clinical study.
Tee et al., 2025 [34]	Outcomes were reported for the aggregate cohort and were not separately extractable for microwave ablation (MWA) and radiofrequency ablation (RFA).
Tamai and Okamura, 2021 [35]	Overlapping cohort with the later Tamai 2025 report retained in the review.

During the final audit, the team identified incomplete acquisition of potentially eligible full texts. Supplementary retrieval recovered eight previously unavailable reports; six met the eligibility criteria and were added to the synthesis. The study database, risk-of-bias assessments, statistical analyses, tables, and figures were then regenerated.

Data Extraction

A long-format dataset was constructed with information recorded separately for each publication and treatment group. Extraction was performed in two stages. First, reported values were transcribed as presented in the source article together with their location in the document, without mathematical transformation. These data were then converted into standardized analytic formats, including event counts with denominators, proportions, means with standard deviations (SDs), and hazard ratios (HRs) with their confidence intervals (CIs) when available.

Each reviewer independently completed the extraction process. When extracted values differed, the original publication was re-examined, and agreement was reached by discussion or, if necessary, consultation with a third reviewer. Numerical consistency was additionally checked by comparing reported percentages with the corresponding events and denominators. Extracted variables included publication characteristics, study design, recruitment period, diagnostic criteria, liver function, tumor number and size, anatomic location, baseline alpha-fetoprotein, primary or recurrent treatment status, treatment approach, device characteristics, follow-up, and efficacy and safety outcomes. The extraction domains are presented in Table 3.

**Table 3:** Data extraction framework

Domain	Extracted variables
Study identification	Author and year; country; journal; language; study design; number of centers; and recruitment period.
Population	Hepatocellular carcinoma diagnostic method; tumor stage; liver function; cirrhosis; etiology; and number of tumors.
Tumor characteristics	Tumor size; perivascular or subcapsular location; baseline alpha-fetoprotein concentration; and primary or recurrent treatment status.
Intervention and comparator	Treatment-arm modality; number of participants per arm; treatment approach; ablation system; and device characteristics.
Efficacy outcomes	Initial complete ablation; local tumor progression; intrahepatic distant recurrence; extrahepatic recurrence; overall survival; and recurrence-free survival.
Safety outcomes	Major complications; minor complications; and periprocedural mortality.
Procedure and follow-up	Number of ablation sessions; procedure duration; hospital stay; and follow-up duration.

The prespecified outcomes and corresponding effect measures are presented in Table 4.

**Table 4:** Outcomes and corresponding effect measures

Category	Outcome	Effect measure
Efficacy	Initial complete ablation	Risk ratio (RR)
Efficacy	Local tumor progression	RR
Efficacy	Intrahepatic distant recurrence	RR
Efficacy	Extrahepatic recurrence	RR
Efficacy	Overall survival	Hazard ratio (HR)
Efficacy	Recurrence-free survival	HR
Safety	Major complications	RR
Safety	Minor complications	RR
Safety	Periprocedural mortality	RR

Risk of Bias Assessment

Randomized controlled trials were evaluated with the revised Cochrane Risk of Bias tool (RoB 2), whereas comparative nonrandomized studies were evaluated with the Risk of Bias in Non-randomized Studies of Interventions (ROBINS-I) tool [36,37]. Domain-level and overall judgments were assigned independently by two reviewers. For nonrandomized comparisons, particular attention was given to confounding by indication because allocation to MWA or RFA could have reflected tumor characteristics, technical accessibility, available technology, treatment era, or operator preference.

Statistical Analysis

Pooled effects were estimated under a random-effects framework. The between-study variance component was obtained using restricted maximum likelihood, while uncertainty around the pooled estimates incorporated the Hartung-Knapp-Sidik-Jonkman adjustment [38]. For dichotomous endpoints, treatment effects were expressed as Mantel-Haenszel risk ratios (RRs); time-to-event data were synthesized as hazard ratios using inverse-variance weighting. Heterogeneity across studies was characterized using I² together with the estimated between-study variance, with prediction intervals reported when informative.

Subgroup analyses were performed by study design. Meta-regression including 26 studies assessed mean tumor size as a study-level modifier of local tumor progression. Small-study effects were examined with contour-enhanced funnel plots and Egger regression when at least 10 studies contributed to an outcome [39]. Analyses were conducted in R using the meta and metafor packages (The R Core Team, R Foundation for Statistical Computing, Vienna, Austria) [40,41].

Certainty of Evidence

Certainty was assessed separately for each outcome with the GRADE framework [42]. In accordance with GRADE methodology, randomized evidence entered the assessment at high certainty and nonrandomized evidence at low certainty. Outcome-specific ratings were subsequently downgraded when limitations related to bias, inconsistency, indirectness, imprecision, or publication bias were identified.

Results

Study Selection and Characteristics

The electronic search yielded 4,496 records: 1,800 from PubMed/MEDLINE, 1,450 from Web of Science, 989 from Scopus, and 257 from SciELO and LILACS. Before screening, 1,071 duplicates and 1,102 records without an abstract were removed. The remaining 2,323 records underwent title-and-abstract review, and 2,261 were excluded. Sixty-two reports identified through database screening and one report identified through citation searching were sought for retrieval. Four reports could not be obtained, leaving 59 full-text reports for eligibility assessment. Twenty-one were excluded: 16 secondary, nonprimary, or preclinical reports; three reports without an eligible direct MWA-RFA comparison; one report without outcomes separable by treatment arm; and one duplicate or overlapping publication. Thirty-eight studies met the eligibility criteria. The complete selection process, database breakdown, and exclusion categories are shown in Figure 1.

**Figure 1:**
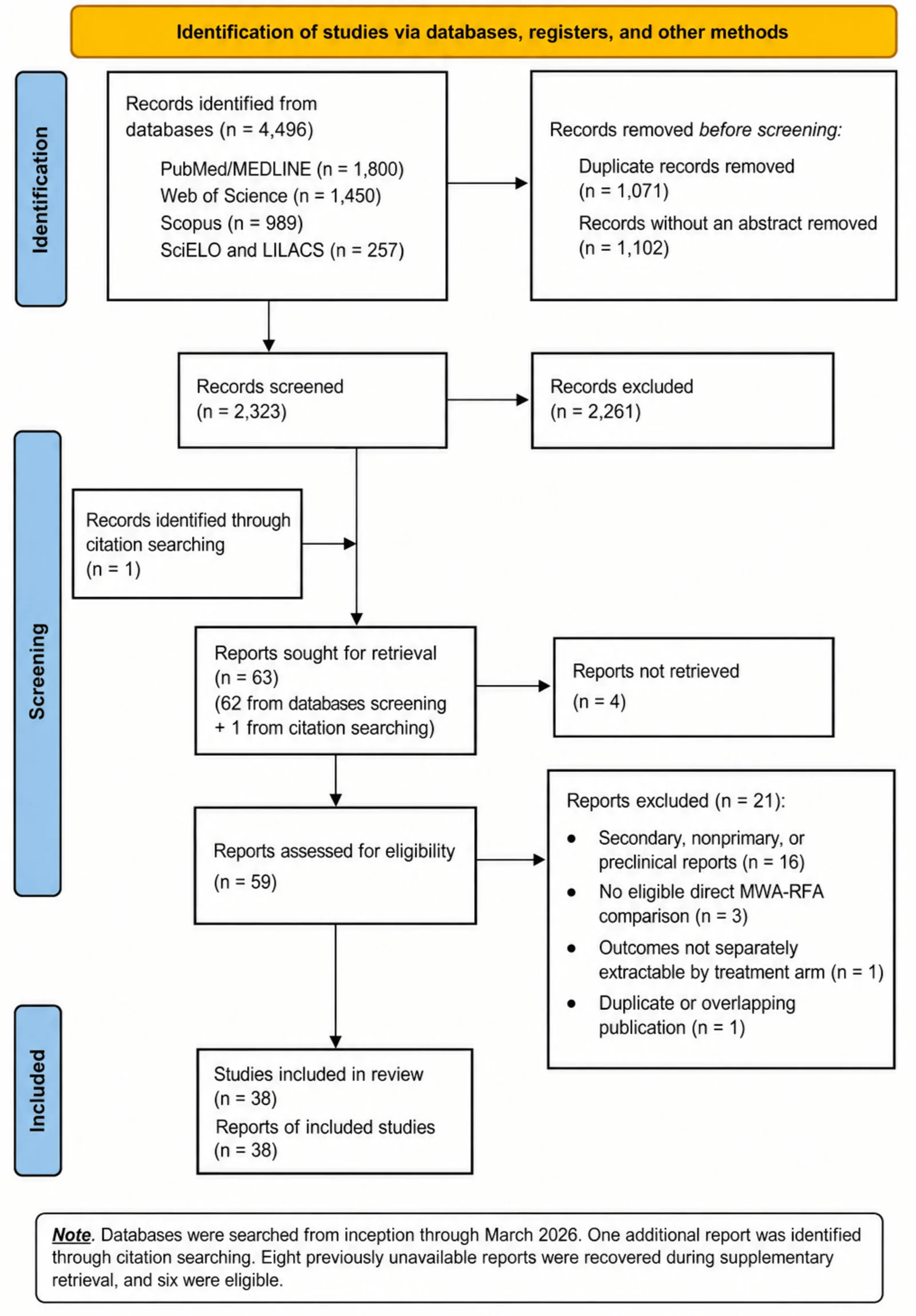
PRISMA flow diagram of the study selection process The search identified 4,496 records from PubMed/MEDLINE (n=1,800), Web of Science (n=1,450), Scopus (n=989), and SciELO and LILACS (n=257). After removal of 1,071 duplicate records and 1,102 records without an abstract, 2,323 records were screened. Sixty-two reports from database screening and one report from citation searching were sought; four could not be retrieved. Fifty-nine full-text reports were assessed; 21 were excluded with reasons, and 38 studies were included. Eight previously unavailable reports were recovered during supplementary retrieval, and six were eligible. PRISMA: Preferred Reporting Items for Systematic Reviews and Meta-Analyses; MEDLINE: Medical Literature Analysis and Retrieval System Online; LILACS: Latin American and Caribbean Health Sciences Literature; MWA: microwave ablation; RFA: radiofrequency ablation

The 38 studies were published between 2005 and 2026 and contributed 8,347 participants in the analytic cohorts: 3,625 treated with MWA and 4,722 treated with RFA [43-80]. Seven reports were randomized controlled trials [43-47,60,62], and 31 were comparative nonrandomized studies. Most nonrandomized studies were retrospective cohorts; several used propensity score matching, and one used inverse probability of treatment weighting. The included studies and their risk-of-bias ratings are summarized in Table 5.

**Table 5:** Characteristics of the included studies IPTW: inverse probability of treatment weighting; MWA: microwave ablation; NR: not reported; PSM: propensity score matching; RCT: randomized controlled trial; RFA: radiofrequency ablation; RoB 2: revised Cochrane Risk of Bias tool; ROBINS-I: Risk of Bias in Non-randomized Studies of Interventions.

Study	Country	Journal	Design	Participants: MWA/RFA	Size (cm)	Follow-up (mo)	Risk of bias
Lu M, 2005 [52]	China	J Gastroenterol	Retrospective cohort	49/53	2.5	25.1	Critical (ROBINS-I)
Ding J, 2013 [53]	China	Eur J Radiol	Retrospective cohort	113/85	2.55	18.32	Serious (ROBINS-I)
Zhang L, 2013 [54]	China	PLoS ONE	Retrospective cohort	77/78	2.2	24.5	Serious (ROBINS-I)
Abdelaziz A, 2014 [43]	Egypt	Surg Endosc	RCT	66/45	2.9	27	High (RoB 2)
Cillo U, 2014 [50]	Italy	HPB	Prospective cohort (PSM)	28/28	2.5	24	Moderate (ROBINS-I)
Chinnaratha M, 2015 [55]	Australia	Eur J Gastroenterol Hepatol	Retrospective cohort	25/101	2.15	13.5	Serious (ROBINS-I)
Vogl T, 2015 [56]	Germany	Abdom Imaging	Retrospective cohort	28/25	3.6	NR	Serious (ROBINS-I)
Potretzke T, 2016 [51]	USA	J Vasc Interv Radiol	Retrospective cohort	99/55	2.2	24	Serious (ROBINS-I)
Lee K, 2017 [57]	Hong Kong (China)	Asian J Surg	Retrospective cohort	26/47	3.75	47.5	Serious (ROBINS-I)
Santambrogio R, 2017 [58]	Italy	Ann Surg Oncol	Retrospective cohort	60/94	2.15	31	Serious (ROBINS-I)
Xu, 2017 [59]	China	Chin J Cancer	Retrospective cohort	301/159	1.7	53.0	Moderate (ROBINS-I)
Yu J, 2017 [60]	China	Gut	RCT	203/200	2.7	35.2	Some concerns (RoB 2)
Liu W, 2018 [61]	China	Aliment Pharmacol Ther	Retrospective cohort (PSM)	126/436	2.25	36.8	Moderate (ROBINS-I)
Vietti Violi N, 2018 [44]	France/Switzerland	Lancet Gastroenterol Hepatol	RCT	71/73	1.8	26	Some concerns (RoB 2)
Kamal A, 2019 [62]	Egypt	J Gastrointest Oncol	RCT	28/28	3.25	12	Some concerns (RoB 2)
Bouda D, 2020 [63]	France	Cardiovasc Intervent Radiol	Retrospective cohort (PSM)	79/70	2.13	34	Moderate (ROBINS-I)
Chong C, 2020 [45]	Hong Kong (China)	HPB	RCT	47/46	3.1	38.3	Low (RoB 2)
Du S, 2020 [64]	China	PLoS ONE	Retrospective cohort (PSM)	218/234	2.9	28.1	Moderate (ROBINS-I)
McDevitt J, 2020 [65]	USA	Clinical Imaging	Retrospective cohort	40/70	NR	17.2	Moderate (ROBINS-I)
Suwa K, 2020 [66]	Japan	Mol Clin Oncol	Retrospective cohort	44/55	1.72	7.8	Serious (ROBINS-I)
An C, 2021 [48]	China	Eur Radiol	Retrospective cohort (IPTW)	74/70	2.0	38.2	Serious (ROBINS-I)
Feng Y, 2021 [49]	China	HPB	Retrospective cohort (PSM)	63/107	2.8	43	Moderate (ROBINS-I)
Han X, 2021 [67]	China	Abdom Radiol	Retrospective cohort	51/150	2.21	36.7	Serious (ROBINS-I)
Kuroda H, 2021 [68]	Japan	Cancers	Retrospective cohort (PSM)	150/150	2.68	13.7	Moderate (ROBINS-I)
Suwa K, 2021 [69]	Japan	Abdom Radiol	Retrospective cohort (PSM)	72/72	1.77	10.9	Moderate (ROBINS-I)
Lee S, 2022 [70]	South Korea	J Clin Med	Retrospective cohort	50/100	2.1	25.3	Moderate (ROBINS-I)
Shen Q, 2022 [71]	China	J Cancer Res Ther	Retrospective cohort	44/34	2.48	37.6	Serious (ROBINS-I)
Zheng H, 2022 [72]	China	Eur Radiol	Retrospective cohort (PSM)	69/69	2.5	58	Moderate (ROBINS-I)
Wang X, 2023 [73]	China	Eur J Radiol	Retrospective cohort (PSM)	75/137	2.0	54	Moderate (ROBINS-I)
Vogl T, 2024 [46]	Germany	Fortschr Röntgenstr	RCT	25/25	2.33	42.7	Some concerns (RoB 2)
Xiong Y, 2024 [74]	China	Transl Cancer Res	Retrospective cohort (PSM)	268/268	3.0	48.6	Moderate (ROBINS-I)
Cheung W, 2025 [78]	Hong Kong (China)	HPB	Retrospective cohort	57/211	2.3	NR	Serious (ROBINS-I)
Sugimoto K, 2025 [47]	Japan	JHEP Rep	RCT	119/117	1.9	33	Some concerns (RoB 2)
Tamai H, 2025 [75]	Japan	Hepatol Res	Retrospective cohort	239/71	2.0	43.1	Serious (ROBINS-I)
Yang W, 2025 [76]	China	Int J Hyperthermia	Retrospective cohort (PSM)	266/762	1.81	NR	Moderate (ROBINS-I)
Yu H, 2025 [77]	China	HPB	Retrospective cohort (PSM)	74/74	2.4	40	Moderate (ROBINS-I)
Bahloul C, 2026 [79]	France	World J Gastroenterol	Retrospective cohort	120/242	NR	27	Moderate (ROBINS-I)
Kim J, 2026 [80]	South Korea	Korean J Radiol	Retrospective cohort (PSM)	81/81	1.5	40.0	Moderate (ROBINS-I)

Among randomized trials, one was rated at low overall risk of bias, five raised some concerns, and one was rated at high risk. Among nonrandomized studies, 17 were rated at moderate risk, 13 at serious risk, and one at critical risk. Confounding was the most frequent reason for serious or critical judgments.

Initial Complete Ablation

Twenty-eight studies involving 6,036 participants reported initial complete ablation. The pooled comparison showed no clinically meaningful difference between MWA and RFA (RR 1.01, 95% CI 1.00-1.02; I²=0%). Study-specific and pooled estimates are displayed in Figure 2.

**Figure 2:**
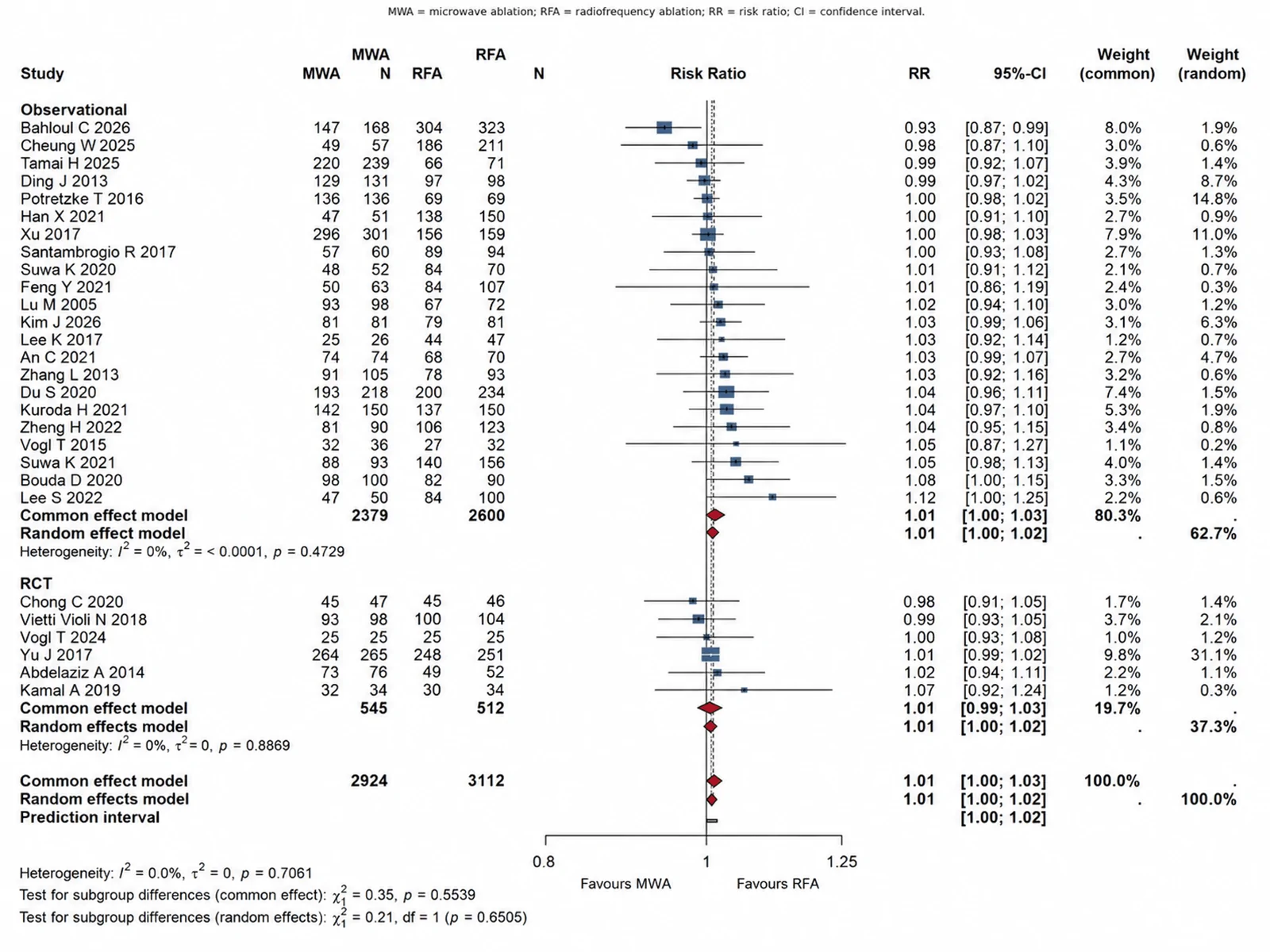
Initial complete ablation: MWA versus RFA Forest plot comparing initial complete ablation after microwave ablation (MWA) and radiofrequency ablation (RFA). The pooled random-effects risk ratio (RR) was 1.01 (95% confidence interval (CI) 1.00-1.02; I²=0%). Included studies: Abdelaziz et al. [43], Vietti Violi et al. [44], Chong et al. [45], Vogl et al. [46], An et al. [48], Feng et al. [49], Potretzke et al. [51], Lu et al. [52], Ding et al. [53], Zhang et al. [54], Vogl et al. [56], Lee et al. [57], Santambrogio et al. [58], Xu et al. [59], Yu et al. [60], Kamal et al. [62], Bouda et al. [63], Du et al. [64], Suwa et al. [66], Han et al. [67], Kuroda et al. [68], Suwa et al. [69], Lee et al. [70], Zheng et al. [72], Tamai et al. [75], Cheung et al. [78], Bahloul et al. [79], and Kim et al. [80].

Local Tumor Progression

Twenty-seven studies involving 5,166 participants reported local tumor progression. MWA was associated with a lower risk than RFA (RR 0.67, 95% CI 0.54-0.83; I²=54%). The association was present in both design strata: randomized controlled trials yielded RR 0.55 (95% CI 0.40-0.76; I²=0%), and comparative nonrandomized studies yielded RR 0.71 (95% CI 0.55-0.91; I²=59%). The design-stratified analysis is shown in Figure 3.

**Figure 3:**
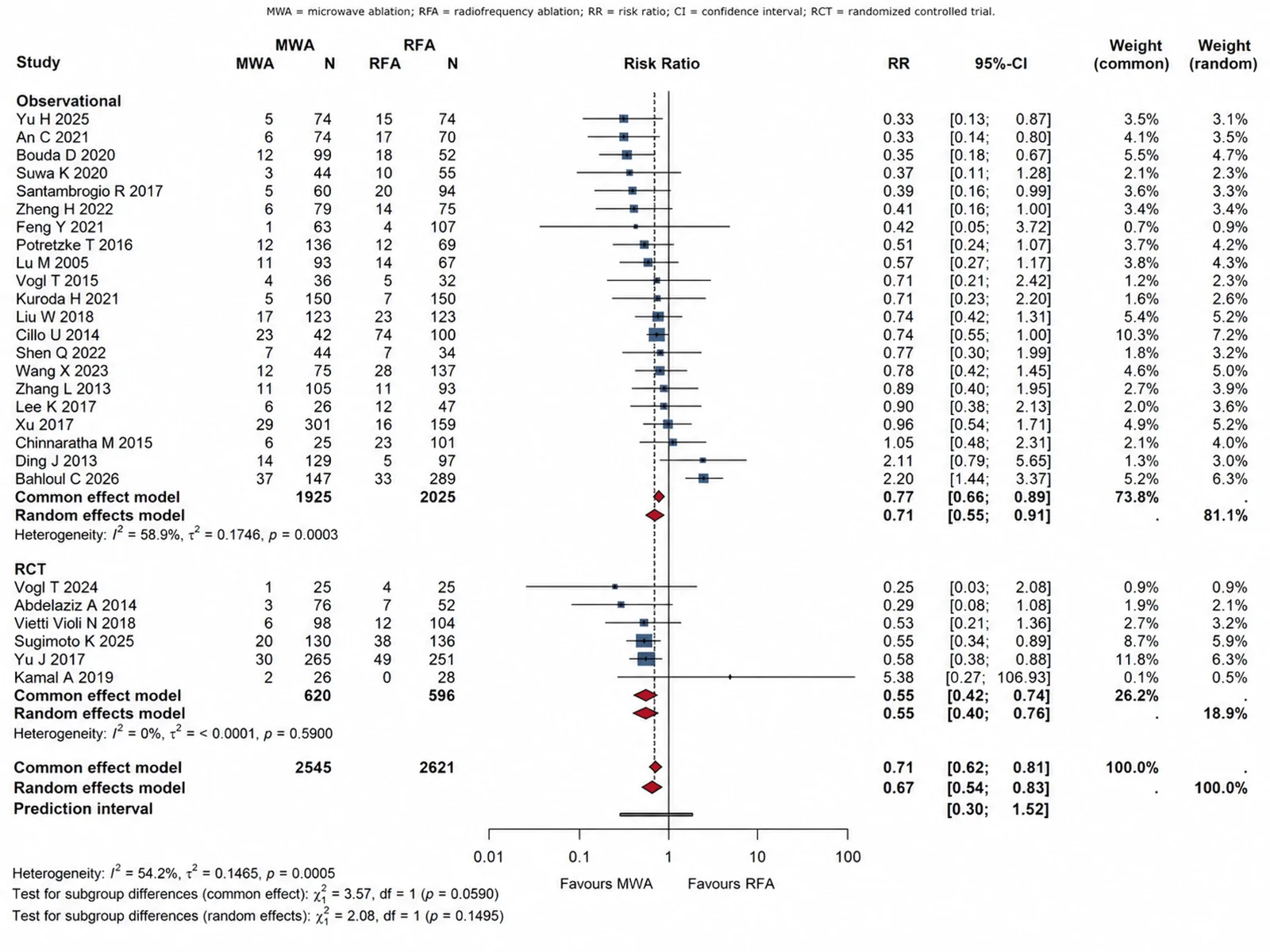
Local tumor progression according to the study design Forest plot comparing local tumor progression after microwave ablation (MWA) and radiofrequency ablation (RFA), stratified by comparative nonrandomized studies and randomized controlled trials (RCTs). The overall random-effects risk ratio (RR) was 0.67 (95% confidence interval (CI) 0.54-0.83; I²=54%). Included studies: Abdelaziz et al. [43], Vietti Violi et al. [44], Vogl et al. [46], Sugimoto et al. [47], An et al. [48], Feng et al. [49], Cillo et al. [50], Potretzke et al. [51], Lu et al. [52], Ding et al. [53], Zhang et al. [54], Chinnaratha et al. [55], Vogl et al. [56], Lee et al. [57], Santambrogio et al. [58], Xu et al. [59], Yu et al. [60], Liu et al. [61], Kamal et al. [62], Bouda et al. [63], Suwa et al. [66], Kuroda et al. [68], Shen et al. [71], Zheng et al. [72], Wang et al. [73], Yu et al. [77], and Bahloul et al. [79].

Intrahepatic Distant Recurrence

Fifteen studies involving 2,740 participants contributed to the analysis of intrahepatic distant recurrence. The pooled RR was 0.89 (95% CI 0.79-1.00; I²=45%). Although the point estimate favored MWA, the result did not establish a conclusive between-technique difference. The forest plot is presented in Figure 4.

**Figure 4:**
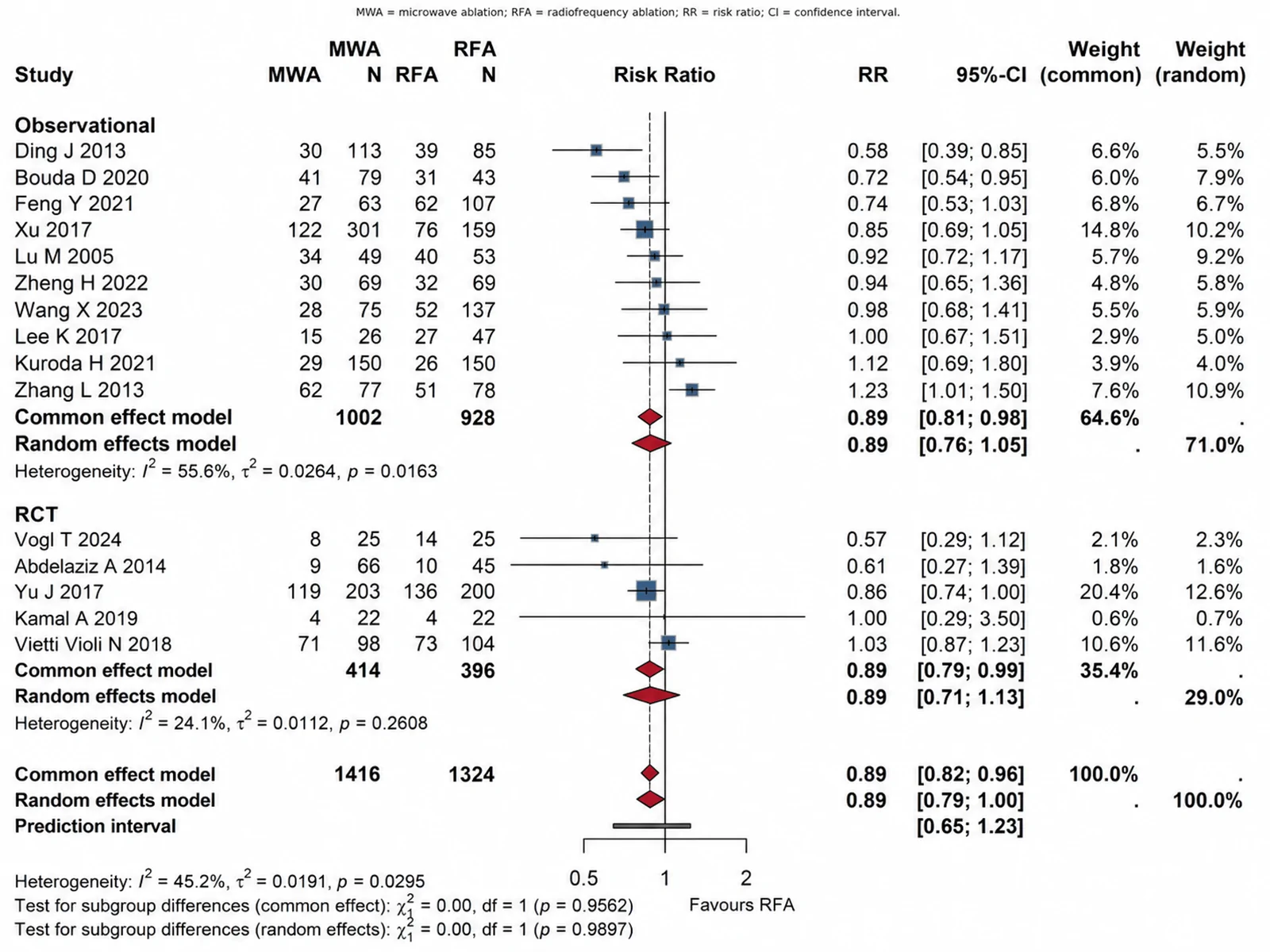
Intrahepatic distant recurrence: MWA versus RFA Forest plot comparing intrahepatic distant recurrence after microwave ablation (MWA) and radiofrequency ablation (RFA). The pooled random-effects risk ratio (RR) was 0.89 (95% confidence interval (CI) 0.79-1.00; I²=45%). Included studies: Abdelaziz et al. [43], Vietti Violi et al. [44], Vogl et al. [46], Feng et al. [49], Lu et al. [52], Ding et al. [53], Zhang et al. [54], Lee et al. [57], Xu et al. [59], Yu et al. [60], Kamal et al. [62], Bouda et al. [63], Kuroda et al. [68], Zheng et al. [72], and Wang et al. [73].

Survival Outcomes

Only three studies reported usable HRs for overall survival and three for recurrence-free survival. The pooled HR was 1.12 for overall survival (95% CI 0.32-3.88; I²=66%) and 0.87 for recurrence-free survival (95% CI 0.38-1.99; I²=57%). Both estimates were imprecise because few studies provided comparable time-to-event data.

Major and Minor Complications

Twenty-six studies involving 5,493 participants contributed events to the meta-analysis of major complications. The pooled estimate did not demonstrate a conclusive difference (RR 1.12, 95% CI 0.87-1.45; I²=17%). Studies with zero events in both groups are displayed for completeness but do not contribute to the pooled ratio. The study-level results are shown in Figure 5.

**Figure 5:**
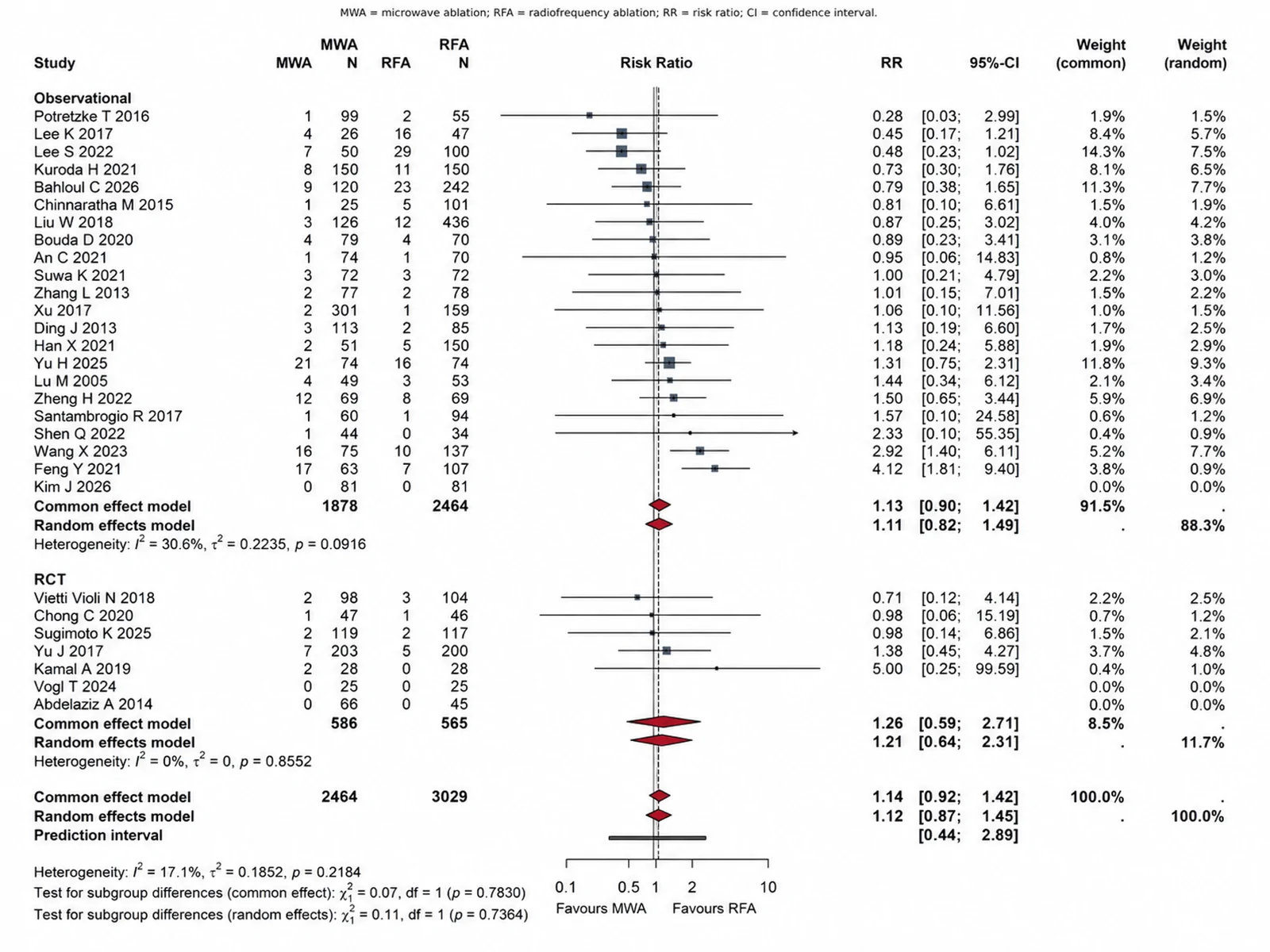
Major complications: MWA versus RFA Forest plot comparing major complications after microwave ablation (MWA) and radiofrequency ablation (RFA). The pooled random-effects risk ratio (RR) was 1.12 (95% confidence interval (CI) 0.87-1.45; I²=17%). Studies displayed in the plot: Abdelaziz et al. [43], Vietti Violi et al. [44], Chong et al. [45], Vogl et al. [46], Sugimoto et al. [47], An et al. [48], Feng et al. [49], Potretzke et al. [51], Lu et al. [52], Ding et al. [53], Zhang et al. [54], Chinnaratha et al. [55], Lee et al. [57], Santambrogio et al. [58], Xu et al. [59], Yu et al. [60], Liu et al. [61], Kamal et al. [62], Bouda et al. [63], Han et al. [67], Kuroda et al. [68], Suwa et al. [69], Lee et al. [70], Shen et al. [71], Zheng et al. [72], Wang et al. [73], Yu et al. [77], Bahloul et al. [79], and Kim et al. [80]. Studies with no events in either treatment arm are shown but do not contribute to the pooled RR.

Fifteen studies involving 3,320 participants reported minor complications. The pooled RR was 1.16 (95% CI 0.96-1.38; I²=54%), without a conclusive difference between techniques. The corresponding forest plot is shown in Figure 6.

**Figure 6:**
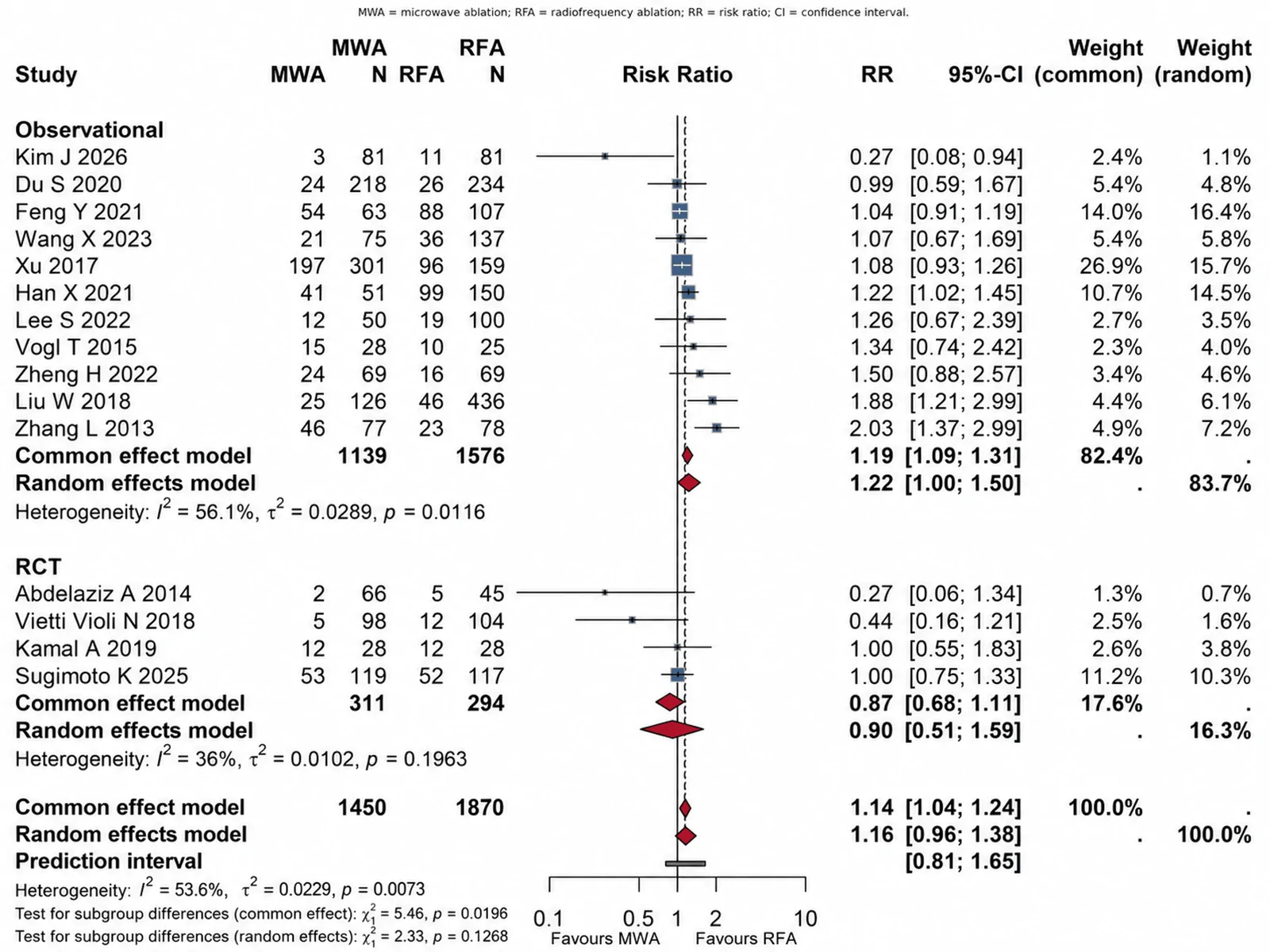
Minor complications: MWA versus RFA Forest plot comparing minor complications after microwave ablation (MWA) and radiofrequency ablation (RFA). The pooled random-effects risk ratio (RR) was 1.16 (95% confidence interval (CI) 0.96-1.38; I²=54%). Included studies: Abdelaziz et al. [43], Vietti Violi et al. [44], Sugimoto K et al. [47], Feng et al. [49], Zhang et al. [54], Vogl et al. [56], Xu et al. [59], Liu et al. [61], Kamal et al. [62], Du et al. [64], Han et al. [67], Lee et al. [70], Zheng et al. [72], Wang et al. [73], and Kim et al. [80].

Periprocedural Mortality

Periprocedural death was uncommon. Four studies contributed events to the pooled analysis, producing an RR of 1.47 (95% CI 0.25-8.76; I²=0%). The wide interval reflects the small number of events and prevents a precise comparative safety estimate.

Meta-Regression, Small-Study Effects, and Certainty

Meta-regression did not identify mean tumor size as a statistically significant modifier of the local tumor progression effect (coefficient 0.12; P=.519). The fitted association and confidence band are presented in Figure 7.

**Figure 7:**
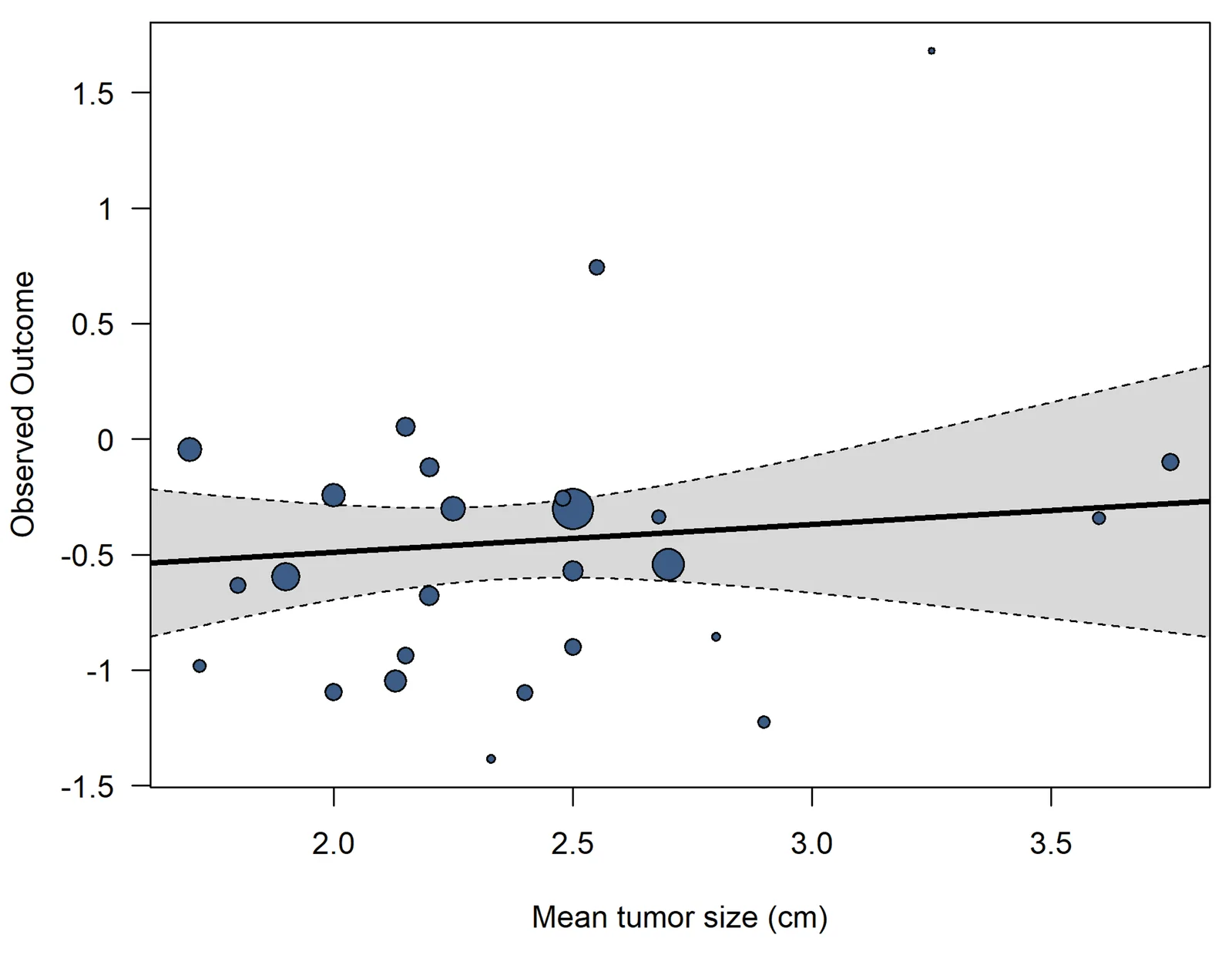
Meta-regression of local tumor progression according to mean tumor size Meta-regression of the logarithmic risk ratio for local tumor progression according to mean tumor size, including 26 studies. Each circle represents a study, and circle area is proportional to study weight. The solid line represents the fitted meta-regression relationship, and the shaded band represents the 95% confidence interval. The regression coefficient was 0.12 (P=.519), indicating that mean tumor size was not a statistically significant study-level modifier of the association between microwave ablation and radiofrequency ablation for local tumor progression.

Contour-enhanced funnel plots showed no marked visual asymmetry. Egger regression was nonsignificant for the five outcomes with at least 10 contributing studies. The funnel plot for local tumor progression is presented in Figure 8.

**Figure 8:**
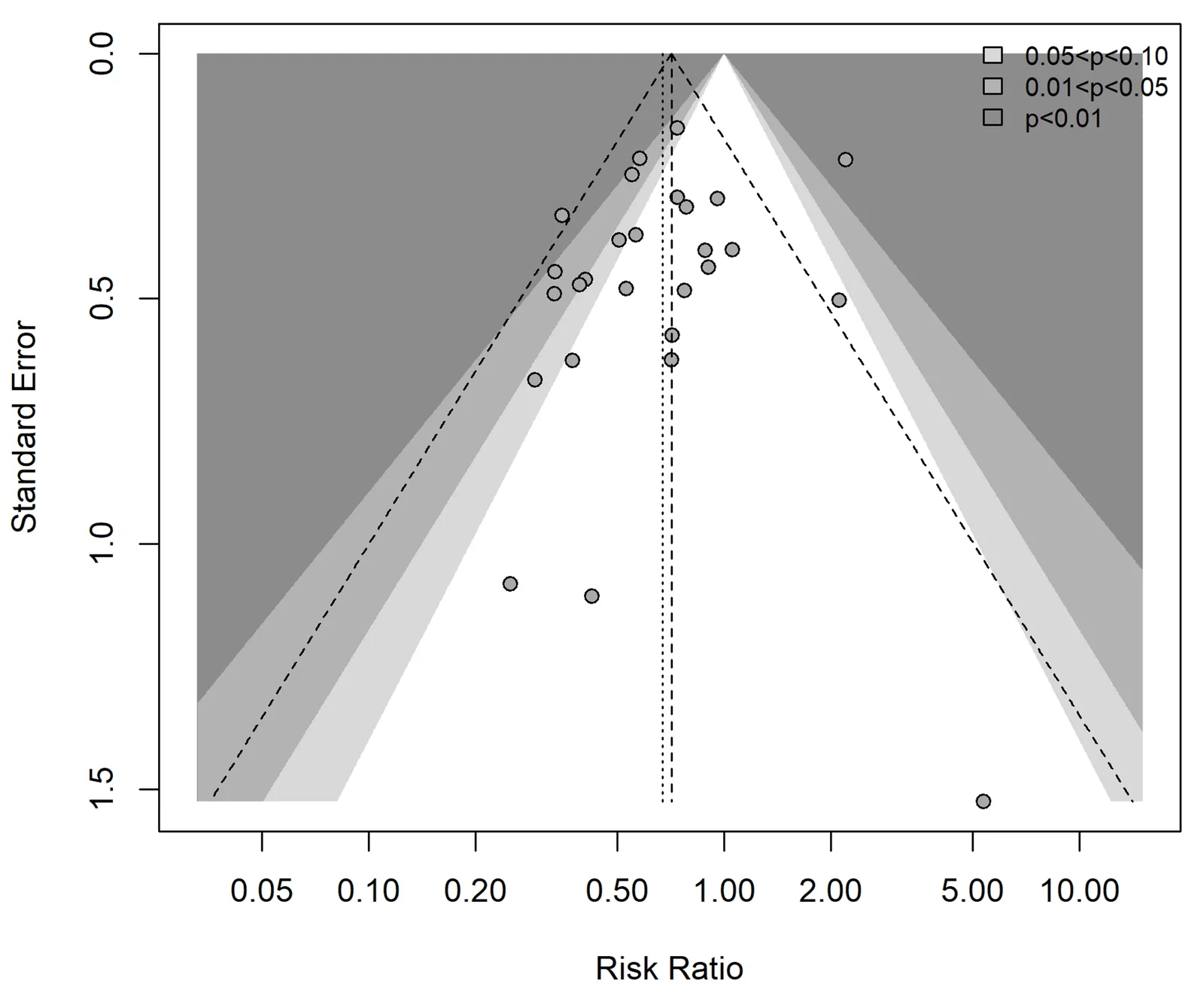
Contour-enhanced funnel plot assessing small-study effects for local tumor progression. Each point represents an individual study. The vertical line represents the pooled effect estimate, while the shaded contours indicate regions of statistical significance. The distribution of studies showed no marked visual asymmetry, providing no clear evidence of small-study effects for this outcome.

The certainty assessment was low for local tumor progression and very low for all remaining outcomes. The pooled estimates, absolute effects, heterogeneity, certainty ratings, and clinical interpretations are summarized in Table 6.

**Table 6:** Summary of findings and certainty of evidence CI: confidence interval; GRADE: Grading of Recommendations Assessment, Development and Evaluation; HR: hazard ratio; MWA: microwave ablation; NRSI: nonrandomized study of an intervention; RCT: randomized controlled trial; RFA: radiofrequency ablation; RR: risk ratio.

Outcome	Number of studies (design)	Patients	Relative effect (95% CI)	Absolute effect (per 1000)	I²	Certainty (GRADE)	Interpretation
Initial complete ablation	28 (6 RCTs, 22 NRSIs)	6036	RR 1.01 (1.00 to 1.02)	RFA 920, MWA 927 (+8)	0%	Very low	No clinically relevant difference
Local tumor progression	27 (6 RCT, 21 NRSI)	5166	RR 0.67 (0.54 to 0.83)	RFA 167, MWA 112 (−55)	54%	Low	Favors MWA
Intrahepatic distant recurrence	15 (5 RCT, 10 NRSI)	2740	RR 0.89 (0.79 to 1.00)	RFA 508, MWA 453 (−55)	45%	Very low	Borderline significance
Major complications	26 (5 RCT, 21 NRSI)	5493	RR 1.12 (0.87 to 1.45)	RFA 57, MWA 64 (+7)	17%	Very low	No significant difference
Minor complications	15 (4 RCT, 11 NRSI)	3320	RR 1.16 (0.96 to 1.38)	RFA 295, MWA 340 (+46)	54%	Very low	No significant difference
Periprocedural mortality	4 (1 RCT, 3 NRSI)	4181	RR 1.47 (0.25 to 8.76)	Few events; not estimable	0%	Very low	No significant difference
Overall survival	3 (0 RCT, 3 NRSI)	—	HR 1.12 (0.32 to 3.88)	—	66%	Very low	No significant difference
Recurrence-free survival	3 (0 RCT, 3 NRSI)	—	HR 0.87 (0.38 to 1.99)	—	57%	Very low	No significant difference

Discussion

The overall pattern observed in our analysis agrees with earlier quantitative reviews of MWA and RFA. Those studies generally found little difference in complete ablation or survival, while some identified a signal favoring MWA for control of the treated site [12,13,18,29]. Differences among previous estimates likely reflect variation in study selection, inclusion of observational evidence, ablation technology, tumor characteristics, and statistical methodology. The present analysis extends this literature by incorporating more recent comparative studies, evaluating randomized and nonrandomized evidence separately, and assessing outcome-level certainty using the GRADE framework.

This synthesis of 38 studies and 8,347 participants found that MWA and RFA produced nearly identical initial complete ablation rates, whereas MWA was associated with less local tumor progression. The local-control association remained evident when randomized controlled trials were analyzed separately. In contrast, the available evidence did not show clear differences in intrahepatic distant recurrence, survival, complications, or periprocedural mortality.

Immediate technical success and durability of tumor control should be interpreted as distinct clinical endpoints. Both technologies can eradicate the visible tumor in appropriately selected patients, which helps explain the RR of 1.01 for initial complete ablation. Later progression at the treated site may depend on whether a sufficiently wide and continuous ablation margin was created. MWA reaches high temperatures rapidly and is less dependent on conductive heat transfer, properties that may reduce distortion of the treatment zone near flowing vessels [8-10]. Comparative studies focused on perivascular HCC likewise suggest that this physical advantage can improve local control [48,49].

The magnitude of the observed MWA effect varied across the evidence base. Differences among studies in generator settings, applicator technology, number of antennas or electrodes, energy-delivery time, image guidance, operator practice, and definitions of local progression may partly account for the moderate heterogeneity. Margin assessment was also inconsistent. A tumor can meet imaging criteria for complete response even when the peripheral safety margin is inadequate, and insufficient margins are known to increase later local failure [81].

No conclusive reduction in intrahepatic distant recurrence was observed. This finding is biologically plausible because new tumors elsewhere in the liver are strongly influenced by cirrhosis, ongoing hepatic injury, microscopic multifocal disease, and de novo carcinogenesis rather than by the energy source used for the index lesion [3,15,16,82,83]. Improved control of the treated site should therefore not be expected to eliminate distant intrahepatic recurrence.

The survival analyses were underpowered. Only three studies supplied usable HRs for overall survival and three for recurrence-free survival, and the pooled intervals were wide. Survival in HCC also depends on hepatic functional reserve, cirrhosis decompensation, subsequent tumors, transplantation eligibility, salvage treatment, and comorbid disease [3,4,15,16,82,83]. The absence of a statistically conclusive survival difference should not be interpreted as proof of equivalence.

Major and minor complication rates were also similar. Procedural safety depends heavily on the relationship of the tumor to the bile ducts, bowel, diaphragm, gallbladder, heart, and major vessels. Although MWA can create larger zones over shorter periods, that feature requires careful planning in complex locations. Hydrodissection, artificial ascites, and other displacement techniques may reduce injury to adjacent structures. The choice of energy source should therefore be integrated with anatomy, expected ablation geometry, imaging guidance, operator expertise, and available protective techniques [7,11]. This individualized approach is consistent with the BCLC, Complexity, Uncertainty, Subjectivity, Emotion (CUSE) framework, which emphasizes evidence-based treatment selection while incorporating clinical complexity, technical feasibility, oncologic risk, local expertise and resources, and patient-specific factors [4].

Methodological safeguards of this review included prospective protocol registration, independent duplicate screening and extraction, risk-of-bias tools selected according to study design, Hartung-Knapp-Sidik-Jonkman-adjusted random-effects synthesis, analyses stratified by design, and outcome-specific GRADE evaluation. These strengths should nevertheless be considered alongside several important limitations. Most evidence was nonrandomized and vulnerable to confounding by indication. The literature was concentrated in East Asia, where hepatitis B virus infection was common. Devices, procedural protocols, outcome definitions, and follow-up schedules varied. Reporting of ablation margins, perivascular location, technical parameters, and time-to-event outcomes was incomplete, limiting subgroup and survival analyses. An incomplete full-text acquisition process was identified during the final audit and corrected through documented supplementary retrieval. Finally, certainty was low for local tumor progression and very low for all other outcomes.

Future randomized comparisons should adopt harmonized definitions for complete ablation, local tumor progression, and complications [84]. Detailed reporting of devices and procedural parameters, objective measurement of ablative margins, stratification according to tumor size and vascular proximity, and longer-term time-to-event follow-up would substantially improve comparability across trials. Patient-level data on procedure duration, treatment sessions, hospital stay, quality of life, additional treatments, and cost would help determine whether better local control translates into a meaningful advantage for particular patients.

## Conclusions

In adults with HCC suitable for local ablation, MWA was associated with less local tumor progression than RFA, while initial complete ablation, safety, and survival outcomes were not demonstrably different. The most relevant distinction, therefore, appears to involve control of the treated site, potentially because MWA is less susceptible to the heat-sink effect. Both modalities remain valid first-line options. Selection should account for tumor location, the expected ablation margin, proximity to vulnerable structures, operator experience, procedural safety, device availability, and cost. More definitive comparisons will require randomized studies with sufficient statistical power, harmonized outcome definitions, and extended follow-up of time-to-event endpoints.
